# Tacrolimus (FK506) Attenuates Hepatic Ischemia–Reperfusion Injury via Oxidative Glutathione Metabolism and Suppression of Lipoxygenase-Mediated Cell Death

**DOI:** 10.3390/antiox15050557

**Published:** 2026-04-28

**Authors:** Moritz Drefs, Malte J. Schirren, Dominik T. Koch, Severin J. Jacobi, Michael Neuberger, Lesca M. Holdt, Bernhard W. Renz, Jens Werner, Markus O. Guba, Dionysios Koliogiannis

**Affiliations:** 1 Department of General, Visceral and Transplantation Surgery, LMU University Hospital, Ludwig Maximilian University, 81377 Munich, Germany; moritz.drefs@med.uni-muenchen.de (M.D.); malte.schirren@med.uni-muenchen.de (M.J.S.); dominik.koch@med.uni-muenchen.de (D.T.K.); severin.jacobi@med.uni-muenchen.de (S.J.J.); michael.neuberger@med.uni-muenchen.de (M.N.); bernhard.renz@med.uni-muenchen.de (B.W.R.); jens.werner@med.uni-muenchen.de (J.W.); markus.guba@med.uni-muenchen.de (M.O.G.); 2 Institute of Surgical Research at the Walter Brendel Centre of Experimental Medicine, LMU University Hospital, Ludwig Maximilian University, 81377 Munich, Germany; 3 Institute of Laboratory Medicine, LMU University Hospital, Ludwig Maximilian University, 81377 Munich, Germany; lesca.holdt@med.uni-muenchen.de; 4 Faculty of Medicine, Ludwig Maximilian University, 80539 Munich, Germany; 5 Transplantation Center Munich, LMU University Hospital, Ludwig Maximilian University, 81377 Munich, Germany

**Keywords:** hepatic ischemia–reperfusion injury, tacrolimus, 12/15-lipoxygenase, glutathione peroxidase 4, reactive oxygen species, liver transplantation, programmed cell death, apoptosis, necroptosis, ferroptosis

## Abstract

Background: Hepatic ischemia–reperfusion injury (IRI) remains a major challenge in liver transplantation (LTx) and hepatectomy. Previous studies identified a 12/15-lipoxygenase (12/15-LOX)-driven lipid peroxidation cascade promoting cell death, whereas glutathione peroxidase 4 (GPx4)-dependent metabolism acts antagonistically. This study investigated whether tacrolimus protects against hepatic IRI through this redox axis. Methods: Male *C57BL/6* mice underwent 65% partial hepatic warm ischemia and reperfusion with or without tacrolimus preconditioning. Liver tissue and serum were analyzed by spectral photometry, Western blotting, TUNEL assay, and serum enzyme measurement. Results were statistically analyzed and compared with previously published results of 12/15-LOX inhibition by baicalein pretreatment and its carrier DMSO. Also, the combination of both tacrolimus and baicalein was investigated. Results: Tacrolimus increased the oxidative glutathione activity quotient (GSSG/GSH) by 75.1% (*p* = 0.0302), attenuated MAPK signaling, reduced SAPK/JNK by 84.6% (*p* = 0.0059), with ERK1/2 showing a downward trend, decreased Caspase-3 activation by 66.9% (*p* < 0.001) and PARP cleavage by 59.9% (*p* = 0.0330), and lowered TUNEL-positive cell death by 61.8% (*p* = 0.0015). Tacrolimus achieved hepatoprotection comparable to 12/15-LOX inhibition, but without hepatotoxicity, whereas combined treatment conferred no additional benefit yet bore toxic properties. Conclusions: Tacrolimus preconditioning mitigates hepatic IRI through a glutathione-linked redox–signaling–cell death axis and exerts cytoprotective effects beyond immunosuppression.

## 1. Introduction

Hepatic ischemia–reperfusion injury (IRI) remains a major challenge in liver transplantation (LTx) and major hepatic surgery. It represents an unavoidable yet modifiable cause of postoperative morbidity and mortality [1,2] and also occurs frequently in trauma and shock [3,4]. Pathophysiologically, IRI arises from ATP depletion during ischemia due to anaerobic metabolism [5]. Upon reoxygenation, the abrupt generation of reactive oxygen species (ROS) and various inflammatory mediators leads to cell damage and triggers cell death [6,7,8]. This cell death is classified as a combination of necrosis, apoptosis, intermediate forms such as necroptosis and iron-dependent cell death cascades, known as ferroptosis [9,10,11,12]. Uncontrolled oxidation of membrane-bound fatty acids is a central effector of hepatic IRI, resulting in the decay of both membrane and cell integrity [8,13,14]. Mitochondria have been identified as key initiators and amplifiers of these processes [15].

Among mitochondrial redox enzymes, 12/15-lipoxygenase (12/15-LOX) catalyzes the peroxidation of membrane-bound arachidonic acid under oxidative stress, thereby promoting apoptotic and ferroptotic cell death through ROS accumulation [16,17,18]. In direct opposition, the selenoprotein glutathione peroxidase-4 (GPx4) is the only membrane-bound GPx subvariant capable of detoxifying the 12/15-LOX product 12S/15S-hydroperoxyeicosatetraenoic acid (12S/15S-HpETE), thereby preserving cellular viability [17,19,20]. The relevance of this 12/15-LOX/GPx4-dependent cell metabolism has already been demonstrated in neuronal and myocardial IRI [21,22,23,24,25].

Crucially, GPx4-mediated detoxification of lipid hydroperoxides requires reduced glutathione (GSH) as its obligatory cofactor. Preservation of glutathione homeostasis therefore represents the upstream metabolic prerequisite for maintaining GPx4-dependent control of 12/15-LOX-driven lipid peroxidation, neutralizing lipid hydroperoxides, and preserving mitochondrial integrity [19,20,26,27,28,29]. Despite these insights, the precise mediator pathways linking glutathione metabolism, 12/15-LOX/GPx4 activity and downstream cell death in hepatic IRI remain incompletely understood. Evidence from GPx4-deficient mice models suggests that apoptosis-inducing factor (AIF) may contribute to cell death signaling through modulation of 12/15-LOX activity [20]. This concept is supported by experimental studies showing that glutathione administration attenuates warm ischemia-induced injury [30,31], as well as by evidence that GPx4 exerts a protective role against oxidative stress and IRI-related tissue damage [26,32]. Together, these observations suggest that alterations in the glutathione system are not merely epiphenomena, but may represent an initiating event within the hepatocellular stress response to ischemia and reperfusion.

In this context, our previous work with the potent 12/15-LOX inhibitor baicalein was able to provide a potential crosslink. In that study, pharmacological 12/15-LOX inhibition was associated with upregulation of oxidative glutathione metabolism, attenuation of postischemic cell death, and modulation of several stress- and apoptosis-associated mediator proteins after hepatic IRI [33]. Thus, our earlier findings already indicated that targeting the 12/15-LOX/GPx-dependent redox axis affects not only metabolic peroxide detoxification but also downstream injury signaling, although dose-dependent hepatotoxicity of baicalein limited its translational applicability [33].

Once lipid peroxides accumulate, oxidative stress-responsive signaling pathways are engaged. In particular, the MAPKs SAPK/JNK and ERK1/2 act as stress sensors that propagate injury signals downstream [34,35,36]. Activation of these pathways is followed by engagement of apoptotic execution mechanisms, reflected by cleavage of Caspase-3 and its substrate PARP, both of which have been linked to hepatic IRI-related tissue damage [37,38,39]. Taken together with our previous baicalein data [33], this supports a hierarchical cascade in which preservation of glutathione-dependent peroxide detoxification suppresses stress signaling and thereby limits terminal cell death execution.

Tacrolimus (FK506), a calcineurin inhibitor widely used for immunosuppression after LTx, was first isolated from a fungal culture in 1984 [40,41,42]. Tacrolimus inhibits the intracellular calcineurin–calmodulin complex in T cells and consequently suppresses nuclear interleukin-2 signaling, resulting in effective CD4+ and CD8+ T-cell inactivation [42,43,44,45,46,47,48]. Beyond immunosuppression, tacrolimus exerts anti-inflammatory effects, reduces cytokine release and neutrophil migration, and has shown favorable outcomes in experimental postischemic liver models [49,50,51,52,53,54,55,56,57,58]. In experimental transplantation and post-hepatectomy models, local administration of tacrolimus has additionally been associated with improved glutathione metabolism, suggesting that its hepatoprotective properties may intersect with the 12/15-LOX/GPx4 axis at an upstream metabolic level [59,60].

Based on these observations and on our previous demonstration that 12/15-LOX inhibition prevents ROS-mediated liver cell death through modulation of glutathione metabolism [33], one might hypothesize that tacrolimus preconditioning preserves glutathione-dependent redox metabolism, thereby functionally supporting GPx4-mediated control of 12/15-LOX-derived lipid peroxides, attenuating SAPK/JNK and ERK1/2 activation, and ultimately reducing Caspase-3/PARP cleavage and hepatocellular death after hepatic IRI. The objective of the present study was therefore to evaluate the impact of tacrolimus preconditioning across this mechanistic sequence in a murine wild-type model and to compare its effects with pharmacological 12/15-LOX inhibition.

## 2. Materials and Methods

This study was conducted using a live, single-animal, murine experimental model. Male *C57BL/6* wild-type mice aged 8–10 weeks (Charles River Laboratories, Sulzfeld, Germany) were used. All experiments were approved by the government of Upper Bavaria under the protocol number 55.2.1.54-2532-100-11 and carried out according to the German legislation on the protection of animals. The surgical procedure was adapted from Hori et al. and Mitchel et al. and has been previously established in our laboratory [33,61,62].

Briefly, mice were anesthetized with a combination of midazolam, medetomidine and fentanyl. A polypropylene catheter was inserted retrogradely into the right carotid for continuous monitoring of mean arterial pressure and heart rate and for fluid administration (0.9% saline). Additional fluid substitution was provided via repeated intraperitoneal administration of warm saline solution throughout the operative procedure. After laparotomy, the liver lobes were mobilized and exposed adequately. Approximately 10 min after laparotomy, warm (37 °C) reversible ischemia of the right anterior, left anterior and left posterior liver segment was induced by clamping the common supplying pedicle using a microclip, resulting in an ischemic fraction of 65% of the liver [61]. The respective liver lobes were exposed to a total warm ischemic time of 60 min, followed by removal of the clip and hepatic reperfusion for 90 min. Instability in blood pressure or heart rate determined as irreversible within one minute was defined as exclusion criteria for any animal involved in this study.

At the end of the reperfusion, blood samples were collected from the vena cava under deep anesthesia prior to euthanasia in accordance with common laboratory animal practices and guidelines. The obtained blood was immediately centrifuged at 2000× *g* for 10 min and serum was subsequently stored at −80 °C. Liver tissue from postischemic and non-postischemic lobes was harvested 90 min after reperfusion for histological, Western blot and spectrophotometric analyses.

The present study analyzed six unrandomized groups (*n* = 10 per group; *n* = 60 in total), comprising three IRI groups and three corresponding sham-operated groups. The groups were defined according to the preconditioning treatment administered:

• Control Group: This group did not receive any specific treatment or preconditioning.
• Tacrolimus Group: Tacrolimus (Astellas Pharma, Munich, Germany) pretreatment was initiated 24 h prior to laparotomy. A total volume of 150 µL of solution was administered intraperitoneally. The tacrolimus dose in this case was 300 µg/kg body weight, dissolved in 0.9% saline solution. This corresponded to 75 µg of tacrolimus administered per test animal.
• Combined Tacrolimus + Baicalein Group (TAC/Baicalein): As in the respective individual groups, tacrolimus was administered intraperitoneally (300 µg/kg body weight, dissolved in 0.9% saline solution) 24 h before laparotomy, and intraperitoneal baicalein (Merck, Darmstadt, Germany) pretreatment (120 mg/kg body weight, dissolved in DMSO) was administered 30 min before laparotomy. Application volumes were 150 µL each.

Additionally, comparative analyses were performed with the two groups, for which results have been previously published [30]:

• Baicalein Group: Baicalein pretreatment to inhibit 12/15-lipoxygenase was performed 30 min before laparotomy. For this purpose, 120 mg/kg body weight of baicalein was administered intraperitoneally. This corresponded to an applied amount of approximately 3 mg baicalein per test animal, dissolved in 150 µL dimethyl sulfoxide (DMSO, AppliChem, Darmstadt, Germany) at maximum solubility.
• DMSO Group: The sole effects of the DMSO carrier solution used to dissolve baicalein were investigated in this test group. The solution was administered intraperitoneally 30 min prior to laparotomy. As in the baicalein group, a total volume of 150 µL was administered.

To avoid objectionable interactions caused by a rise in intra-abdominal pressure, the intraperitoneally administered volume did not exceed the limits recommended for laboratory animal care [63,64]. The sham operation included laparotomy, mobilization and exposition of the liver lobes, which were then maintained in this state for the whole surgical duration of the other interventional groups.

The quantification of cells undergoing cell death was conducted in formalin-fixed and paraffin-embedded tissue slices of the hepatic left posterior segment, harvested as previously described. TUNEL staining was performed using an “In Situ Cell Death Detection Kit, POD” (Roche, Basel, Switzerland) in accordance with the manufacturer’s instructions. The process of counter-staining was employed to facilitate the visualization of DNA fragmentation, utilizing the 4′,6-diamidino-2-phenylindole (DAPI) technique (Vector Laboratories, Inc., Newark, CA, USA). The quantification of TUNEL-positive cells was performed by means of planimetric analyses of labeled cells with fluorescence microscopy. The following parameters were utilized: an emission wavelength of 450–500 nm and a fluorescence detection range of 515–565 nm (green light). A total of 10 regions of interest (ROIs) were selected from each tissue slice, with each ROI analyzed separately under 10× enlargement. The microscopic images thus obtained were then processed sequentially using ImageJ (version 1.47, Laboratory for Optical and Computational Instrumentation (LOCI), University of Wisconsin, Madison, WI, USA), thus facilitating automated detection and calculation of the area fraction of TUNEL-positive cells for each group as a direct proportional parameter for dead cell count. All histological examinations were carried out by one of the authors in a blinded manner, while predefined label codes helped group these results adequately hereafter.

The employment of Western blots was instrumental in the detection of proapoptotic protein levels within the liver lobes subjected to IRI. The tissue of the hepatic right anterior segment was minced in a buffer solution that contained 1 µL/mL dithiothreitol (DTT, Sigma-Aldrich, St. Louis, MO, USA) and 10 µL/mL of a protease inhibitor mixture containing aprotinin (100 U/mL, Sigma-Aldrich, USA). The protein concentration in each hepatic tissue lysate was determined using a “PierceTM BCA Protein Assay Kit” (ThermoFisher Scientific, Waltham, MA, USA). After protein extraction and quantification, equal amounts of protein were separated by SDS-PAGE and transferred onto nitrocellulose membranes, followed by overnight incubation with antibodies at 4 °C. Primary antibodies were used to detect phosphorylated (Phospho-SAPK/JNK, Rabbit, 46/54 kDa, 1:1000; Cell Signaling Technology, Danvers, MA, USA) and total stress-activated protein kinase/Jun-amino-terminal kinase (SAPK/JNK, Rabbit, 46/54 kDa, 1:1000; Cell Signaling Technology, USA), phosphorylated (Phospho-ERK1/2, Rabbit, 42/44 kDa, 1:1000; Cell Signaling Technology, USA) and total Mitogen-activated protein kinase p44/42 (ERK1/2, Rabbit, 42/44 kDa, 1:1000; Cell Signaling Technology, USA), Caspase-3 (full-length (Rabbit, 17/19/35 kDa, 1:1000; Cell Signaling Technology, USA) and cleaved forms (Cleaved Caspase-3, Rabbit, 17/19 kDa, 1:1000; Cell Signaling Technology, USA)), and poly-ADP-ribose-polymerase (PARP; full-length and cleaved forms with the same antibody (Rabbit, 89/116 kDa, 1:1000; Cell Signaling Technology, USA)), and GAPDH (rabbit, 37kDa, 1:1000; Cell Signaling Technology, USA) was used as a loading control. For ERK1/2 and SAPK/JNK, phosphorylated and total fractions were assessed on the same membranes by sequential probing with stripping between incubations. Cleaved and full-length forms of Caspase-3 and PARP were likewise analyzed on the same membrane systems, with stripping/reprobing where applicable. Densitometric activity was expressed as the ratio of phosphorylated to total protein for ERK1/2 and SAPK/JNK, and as the ratio of cleaved to full-length protein for Caspase-3 and PARP. All Western blots were processed using a horseradish peroxide (HRP)-linked antibody (anti-rabbit IgG, Cell Signaling Technology, USA) as the secondary antibody. The blotted results were then subjected to visualization by means of the chemiluminescence technique (SuperSignal, ThermoFisher Scientific, USA). Uncropped original blots including all lanes and molecular weight markers are provided in the Supplementary Materials, Figure S1.

Activities of serum parameters for liver cell integrity—glutamate dehydrogenase (GLDH), aspartate aminotransferase (AST) and alanine aminotransferase (ALT)—were determined at 37 °C with an automated analyzer (Hitachi 917, Roche-Boehringer, Mannheim, Germany) using standardized test systems (ALAT-FS, ASAT-FS, and GLDH-FS-DGKC, DiaSys, Holzheim, Germany) within the LMU University’s Institute for Laboratory Medicine.

Intracellular concentrations of glutathione (GSH) and its oxidized state glutathione disulfide (GSSG) were analyzed using spectral photometry and a kinetic test first described by Tietze et al. [65]. Tietze’s test was performed on tissue samples of shock-frozen liver lobes that had undergone IRI and were lysed by the addition of perchloric acid and ethylenediaminetetraacetic acid (EDTA). Protein precipitate was then removed from the centrifuged tissue homogenate in succession. In the following procedure, the total glutathione concentration was determined by measuring the velocity of thionitrobenzoeic acid (TNB) accumulation. Spectral photometry was utilized at 412 nm to detect the product of a spontaneous reaction between GSH within the tissue sample (hepatic right anterior segment) and 5,5-dithiobisnitrobenzoeic acid (DTNB), which was added to the standardized reaction. This velocity is directly proportional to the total glutathione concentration (GSH + GSSG) in the sample. The concentration of GSSG could be determined using the same testing mechanism as previously described, but after preventing auto-oxidation of GSH to GSSG by adding N-ethylmaleimide (NEM). As initially described by Tietze and subsequently elaborated upon by Lauterburg et al. [66], NEM demonstrates a high degree of efficacy in the conjugation of GSH. Consequently, the intracellular concentration of GSH can be calculated by subtracting the measured GSSG levels from the total glutathione levels. This technique enables the estimation of both the substrate and product of GSH-metabolizing selenoperoxidases, albeit indirectly. As already described above, GPx4 seems to play a prominent role in their production, especially in ischemia–reperfusion settings [20,21,22,24,67].

Statistical analyses were performed using GraphPad Prism 10.5 (GraphPad Software Inc., La Jolla, CA, USA). Exact statistical tests are specified for each analysis in the corresponding figure legends. Homogeneity of variance was assessed using Brown–Forsythe’s and/or Bartlett’s test, as appropriate. Depending on the experimental design and comparison structure, data were analyzed using the respective parametric statistical models followed by the appropriate post hoc multiple-comparison tests, if applicable. That implies that for all pairwise group comparisons, one-way ANOVA and Tukey’s post hoc test were used. For multiple group comparisons and for time-course data, two-way ANOVA with Šídák’s post hoc test was used. Data are presented as mean ± SEM, and *n* denotes biological replicates (individual animals). A *p* value < 0.05 was considered statistically significant. For comparisons including the previously published baicalein and DMSO cohorts [33], the original individual-level data from these experiments were reanalyzed together with the current tacrolimus dataset. These historical comparator cohorts were generated using the same murine strain, ischemia–reperfusion protocol, tissue processing workflow, and endpoint definitions as applied in the present study.

## 3. Results

### 3.1. Effects of Tacrolimus Preconditioning on Macrohemodynamics

**Figure 1:**
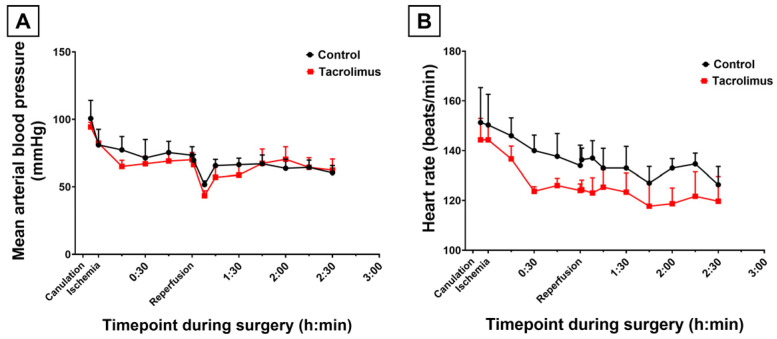
Graphical representation of measured values of macrohemodynamics throughout the course of the experiment inducing ischemia and reperfusion in the liver. Black curves represent the respective values for the control cohort without specific treatment and red curves for the cohort after tacrolimus preconditioning. Intra-arterially measured parameters of mean arterial blood pressure (**A**) and heart rate (**B**) are plotted on the graphs, revealing no statistically significant differences between the two cohorts. However, a non-significant trend towards lowered heart rate after tacrolimus pretreatment is noted. Two-way ANOVA was used for analysis. SEM is presented alongside mean values.

In order to assess whether tacrolimus pretreatment influenced systemic hemodynamics, mean arterial blood pressure (MAP) and heart rate were continuously monitored throughout the experimental procedure via arterial cannulation. No significant differences in MAP were observed between tacrolimus-pretreated mice and untreated controls at any timepoint (Figure 1A). As noted in the control cohort, MAP decreased initially by approximately 30 mmHg after laparotomy and remained stable thereafter. Also, a significant MAP reduction by a maximum of 23 mmHg occurred immediately following reperfusion but returned to baseline within a few minutes (*p* = 0.0073). This reperfusion-associated dip was comparable between groups and consistent with findings from previous studies [33].

Similarly, heart rate values did not differ significantly between the tacrolimus and control groups (Figure 1B). Even though the tacrolimus cohort exhibited slightly lower heart rate values, this difference did not reach statistical significance. Therefore, no animal met the exclusion criteria of this study—neither in this group nor in other groups.

These findings indicate that tacrolimus preconditioning did not alter macrohemodynamic stability, excluding systemic circulatory effects as potential confounders in the subsequent analyses.

### 3.2. Impact of Tacrolimus Preconditioning on Oxidative Glutathione Metabolism

**Figure 2:**
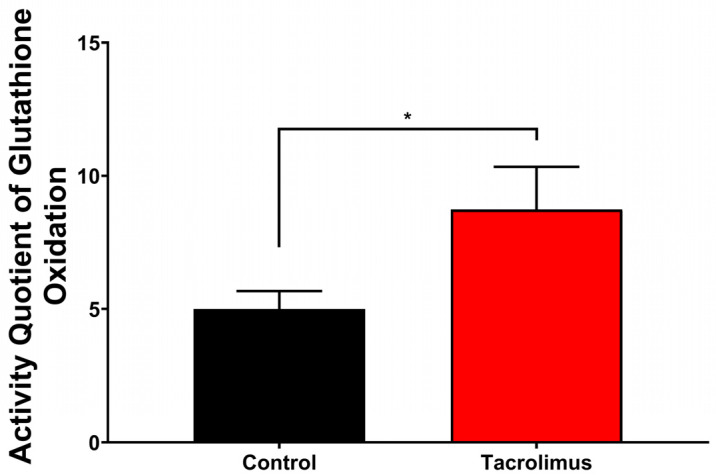
Graphical representation of quantified spectral photometric analyses of intracellular activity of glutathione oxidation within liver tissue (left anterior segment) using Tietze’s kinetic test. The activity quotient of glutathione oxidation is shown and calculated as the ratio of oxidized glutathione disulfide (GSSG) over its reduced substrate glutathione (GSH) in the cohorts after inducing ischemia and reperfusion without pretreatment (black column) and after tacrolimus pretreatment (red column). Tacrolimus preconditioning caused a significant increase in oxidative glutathione metabolism of +75.1% (*p* = 0.0302). One-way ANOVA with Tukey’s post hoc test was used for analysis of all experimental groups. SEM is presented alongside mean values. * *p* < 0.05.

To define the upstream metabolic effects of tacrolimus preconditioning, reduced glutathione (GSH) and its oxidized product glutathione disulfide (GSSG) were quantified in postischemic liver tissue. The GSSG/GSH quotient was used as an indirect readout of oxidative glutathione metabolism. Tacrolimus significantly increased this quotient by 75.1% compared with the untreated control cohort (Figure 2; *p* = 0.0302), indicating enhanced glutathione turnover and consistent with improved glutathione-dependent peroxide detoxification.

Thus, tacrolimus preconditioning significantly enhanced oxidative glutathione metabolism in postischemic liver tissue, consistent with preserved glutathione homeostasis.

### 3.3. Impact of Tacrolimus Preconditioning on Oxidative Stress-Responsive MAPK Signaling and Downstream Apoptotic Execution

**Figure 3:**
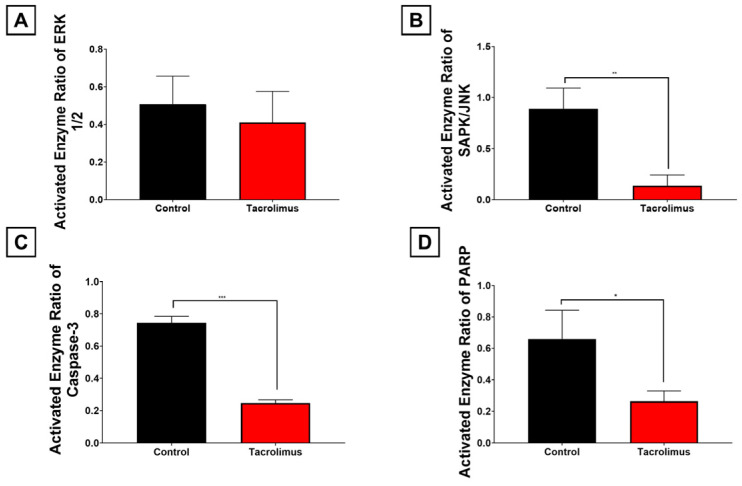
Graphical representation of the activated enzyme ratio of proapoptotic mediator proteins ERK1/2 (Mitogen-activated protein kinase p44/42, 42/44 kDa (**A**)), SAPK/JNK (stress-activated protein kinase/Jun-amino-terminal kinase, 46/54 kDa (**B**)), Caspase-3 (17/19 (cleaved)/35 (total) kDa (**C**)), and PARP (poly-ADP-ribose-polymerase, 89 (cleaved)/116 (total) kDa (**D**)), obtained and subsequently analyzed from liver tissue (right anterior segment) by densitometry after inducing ischemia and reperfusion. The activated enzyme ratio is calculated as the activated subvariant of each protein (=cleaved for PARP and Caspase-3, as well as phosphorylated for ERK1/2 and SPAK/JNK) over the respective total variants. Tacrolimus pretreatment (red column) led to a significant decrease in enzyme activity in SAPK/JNK (−84.6%; *p* = 0.0059; (**B**)), Caspase-3 (−66.9%; *p* < 0.001; (**C**)) and PARP (−59.9%; *p* = 0.0330; (**D**)), as well as to a non-significant negative regulation trend in ERK1/2 (−19.1%; *p* = 0.6717; (**A**)), as compared to the control cohort (black column). One-way ANOVA with Tukey’s post hoc test was used for analysis of all experimental groups. Quantification was based on densitometric analysis of all biological replicates. Full blot images including all lanes and molecular weight markers are provided in Supplementary Figure S1. SEM is presented alongside mean values. * *p* < 0.05; ** *p* < 0.01; *** *p* < 0.001.

Because glutathione-dependent detoxification of lipid peroxides is expected to modulate downstream stress signaling, the activity of oxidative stress-responsive MAPKs and apoptotic execution proteins was analyzed in postischemic liver tissue. These measurements revealed clear differences in enzyme activity between the experimental groups (Figure 3).

As the most proximal signaling mediators examined, the MAPKs ERK1/2 and SAPK/JNK were analyzed first. ERK1/2 activity was calculated as the quotient of the activated, phosphorylated subvariant over its total measurable amount. Although statistical significance was not reached, tacrolimus preconditioning was associated with a notable negative regulatory trend in ERK1/2 activity of −19.1% compared with untreated livers (Figure 3A; *p* = 0.6717).

Tacrolimus treatment also led to a marked −84.6% decrease in stress-activated protein kinase/Jun-amino-terminal kinase (SAPK/JNK) activity, measured as the ratio of activated, phosphorylated SAPK/JNK over its total amount, compared with the untreated control group (Figure 3B). This reduction was highly statistically significant (*p* = 0.0059).

Downstream of MAPK signaling, tacrolimus also reduced Caspase-3 activation, measured as the cleaved fragment relative to total procaspase-3 (Figure 3C). Representative blots showed predominance of the 35 kDa pro-form with markedly reduced appearance of the 17 kDa cleaved fragment in tacrolimus-preconditioned livers. Quantitatively, tacrolimus preconditioning resulted in a statistically significant −66.9% decrease in Caspase-3 activity within the postischemic liver lobes (*p* < 0.001).

Likewise, poly-ADP-ribose polymerase (PARP) cleavage, measured as the cleaved subvariant relative to the full-length protein, was significantly attenuated after tacrolimus pretreatment compared with the control group (Figure 3D; *p* = 0.0330). Representative blots demonstrated preservation of full-length 116 kDa PARP with reduced formation of the 89 kDa cleaved fragment. Overall, PARP activity was reduced by −59.9%.

In summary, these findings indicate that tacrolimus preconditioning suppresses oxidative stress-responsive MAPK signaling and downstream apoptotic execution in postischemic liver tissue.

### 3.4. Effects of Tacrolimus Preconditioning on Hepatic Cell Death After Inducing Ischemia and Reperfusion

**Figure 4:**
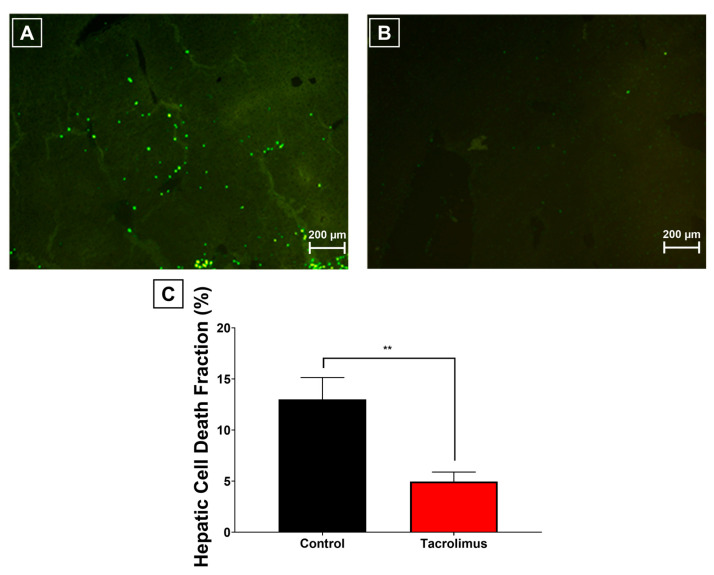
Evaluation of cell death proportion within liver tissue after induction of hepatic ischemia–reperfusion injury. (**A**,**B**): Representative images of fluorescence microscopic detection of decayed cells using TUNEL assays at 10× enlargement of the investigated liver sample (left posterior segment), without pretreatment (**A**) and with tacrolimus preconditioning (**B**). An emission wavelength of 450–500 nm and a fluorescence detection range of 515–565 nm were used. Large representative images with better visibility are shown in Supplementary Figure S2. (**C**): Graphical representation of quantitative planimetric analyses of the hepatic cell death fraction as compared to overall cells. Tacrolimus pretreatment (red column) led to a significant cell death reduction of −61.8% (*p* = 0.0015) compared to the control cohort (black column). One-way ANOVA with Tukey’s post hoc test was used for analysis of all experimental groups. SEM is presented alongside mean values. ** *p* < 0.01.

Consistent with the upstream metabolic and signaling changes, TUNEL assays were performed to quantify the phenotypic consequence at tissue level. Marked differences were observed between untreated controls (Figure 4A) and mice preconditioned with tacrolimus (Figure 4B). Tacrolimus pretreatment significantly attenuated hepatocellular death, reducing the proportion of TUNEL-positive cells by 61.8% compared with the cohort without pretreatment (Figure 4C; *p* = 0.0015).

Accordingly, tacrolimus preconditioning translated the observed redox and signaling effects into substantial protection against ischemia–reperfusion-induced hepatocellular injury.

### 3.5. Effects of Tacrolimus Preconditioning on Serum Liver Enzyme Release

To complement the tissue-based findings and assess potential hepatotoxicity of tacrolimus pretreatment, caval blood samples were analyzed for AST, ALT, and GLDH in both sham-operated and IRI groups. Among sham-operated mice, no relevant toxicity was detected, as tacrolimus did not significantly increase any measured enzyme; instead, slight non-significant reductions were observed for AST (−30.9%; *p* = 0.26), ALT (−33.9%; *p* = 0.33), and GLDH (−6.9%; *p* = 0.82). After IRI, tacrolimus-preconditioned mice again showed lower absolute serum enzyme activities than untreated controls, with relative decreases of −47.0% for AST (*p* = 0.17), −64.6% for ALT (*p* = 0.0062), and −9.7% for GLDH (*p* = 0.77). To account for baseline values under tacrolimus alone, postischemic enzyme levels were additionally compared with those of tacrolimus-treated sham mice. In this within-treatment comparison, IRI still caused significant increases in AST (+300.6%; *p* < 0.001), ALT (+310.8%; *p* < 0.001), and GLDH (+778.5%; *p* = 0.0259).

Comparable increases were also observed in untreated control animals after IRI, with incremental factors of +422.4% for AST (*p* = 0.0423), +667.7% for ALT (*p* = 0.0041), and +805.5% for GLDH (*p* = 0.0200).

**Figure 5:**
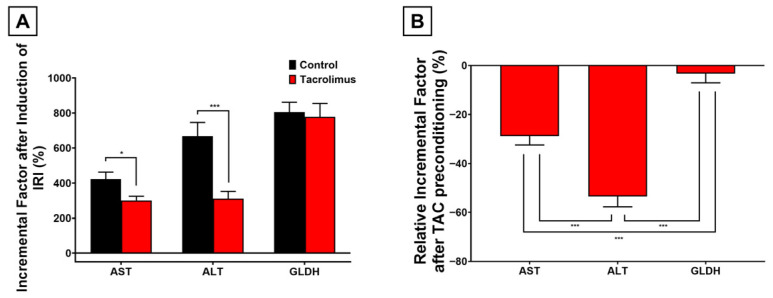
Graphical representation of quantitative serum analyzes for liver enzymes AST (aspartate aminotransferase), ALT (alanine aminotransferase) and GLDH (glutamate dehydrogenase), obtained by caval blood sampling. (**A**): Parameter-specific incremental factors of the respective serum parameters after induction of IRI are shown as a quotient of enzymes measured after ischemia and reperfusion over the same enzyme measured after sham operation, each without pretreatment (black column) and with tacrolimus pretreatment (red column). Two-way ANOVA with Šídák’s post hoc test was used for analysis of all experimental groups. (**B**): Parameter-specific relative incremental factors after tacrolimus pretreatment and hepatic IRI of the respective serum parameters are shown as the relative difference in the respective incremental factors after tacrolimus pretreatment compared to no pretreatment. Tacrolimus pretreatment led to a relatively attenuated increase in liver enzyme activity after inducing IRI, most notably for AST (−28.8%; *p* = 0.0190) and ALT (−53.4%; *p* < 0.001), as compared to no pretreatment. In this context, the ALT increase was attenuated the most, compared to the other enzymes (*p* < 0.001). One-way ANOVA with Tukey’s post hoc test was used for analysis of all experimental groups. SEM is presented alongside mean values. * *p* < 0.05; *** *p* < 0.001.

When these incremental factors were compared between groups, tacrolimus pretreatment attenuated the IRI-induced release of AST by −28.8% (*p* = 0.0190) and ALT by −53.4% (*p* < 0.001), whereas the effect on GLDH was modest (−3.4%; *p* = 0.7811) (Figure 5A). The differences between these relative incremental factors were significant, with AST showing the most pronounced overall involvement (*p* < 0.001 vs. ALT and GLDH) and ALT showing a greater effect than GLDH (*p* < 0.001) (Figure 5B).

### 3.6. Effect of Combined Tacrolimus and Baicalein Pretreatment Across the Investigated Redox-Signaling-Cell Death Cascade

Because baicalein, a potent inhibitor of 12/15-LOX, had previously shown hepatoprotective effects on the same pathway components investigated here [33], we next examined whether combining tacrolimus with baicalein would further strengthen the redox–signaling–cell death cascade outlined above.

**Figure 6:**
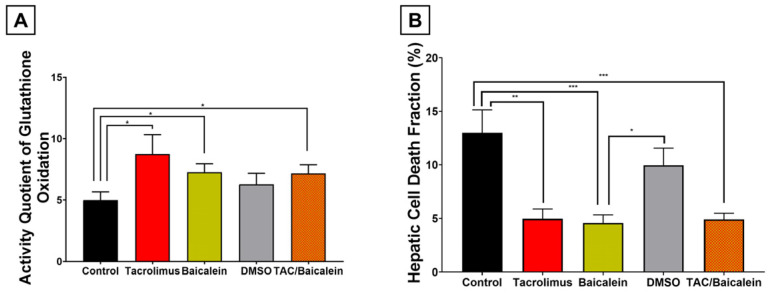
Comparative analyses between different pretreatments with regard to cell death and glutathione oxidation in postischemic livers. (**A**): Graphical representation of quantified spectral photometric analyses of intracellular activity of glutathione oxidation within liver tissue (left anterior segment) as represented by the activity quotient of glutathione oxidation. Preconditioning the liver with tacrolimus (+75.1%; *p* = 0.0302), baicalein (+45.7%; *p* = 0.0329) and the combination of both (+43.7%; *p* = 0.0422) resulted in significantly elevated intrahepatic oxidative glutathione metabolism after ischemia and reperfusion compared to livers that had not undergone pretreatment. However, the results of the three forementioned treatments did not relevantly differ from each other. (**B**): Graphical representation of quantitative planimetric analyses of the hepatic cell death fraction as compared to overall cells after induction of hepatic ischemia–reperfusion injury. Preconditioning the liver with tacrolimus (−61.8%; *p* = 0.0015), baicalein (−74.8%; *p* < 0.001) and the combination of both (−62.3%; *p* < 0.001) resulted in significantly reduced hepatic cell death after ischemia and reperfusion compared to livers that had not undergone pretreatment. In this case as well, the results of the three forementioned treatments did not significantly differ from each other. One-way ANOVA with Tukey’s post hoc test was used for analysis of all experimental groups. SEM is presented alongside mean values. * *p* < 0.05; ** *p* < 0.01; *** *p* < 0.001.

At the upstream metabolic level, combined treatment with tacrolimus and baicalein significantly increased oxidative glutathione metabolism within postischemic liver tissue by +43.7% compared with the control cohort (Figure 6A; *p* = 0.0422). Compared with tacrolimus preconditioning alone, however, this effect was 17.9% lower and therefore represented only a non-significant negative regulatory trend (*p* = 0.2431).

**Figure 7:**
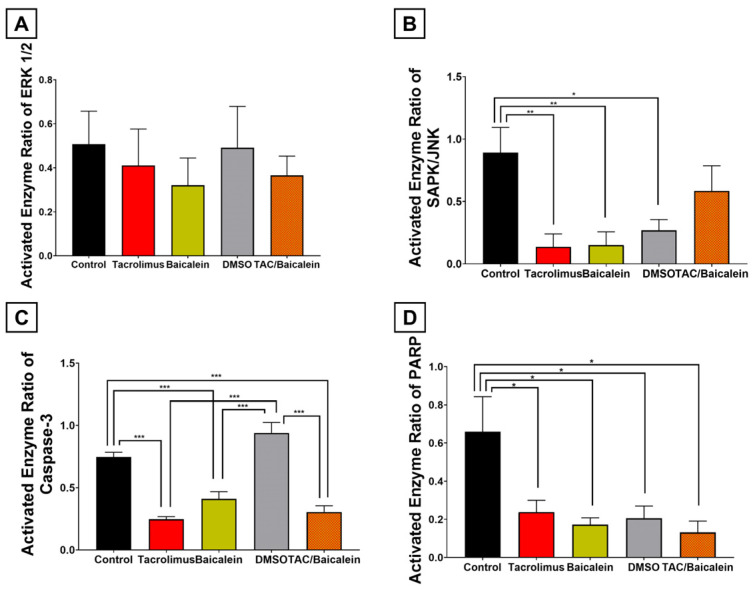
Comparative analyses between different pretreatments with regard to proapoptotic mediator proteins. Graphical representation of the activated enzyme ratio of proapoptotic mediator proteins ERK1/2 (Mitogen-activated protein kinase p44/42 (**A**)), SAPK/JNK (stress-activated protein kinase/Jun-amino-terminal kinase (**B**)), Caspase-3 (**C**), and PARP (poly-ADP-ribose-polymerase (**D**)), obtained and subsequently analyzed via Western blot from liver tissue (right anterior segment) after inducing ischemia and reperfusion. All applied pretreatments resulted in a significant reduction in PARP activation (**D**). A similar result can be regarded for SAPK/JNK, yet without reaching statistical significance, for the combination of tacrolimus and baicalein (**B**). A negative regulatory trend (without significance) is observed after tacrolimus, baicalein and the combined preconditioning for ERK1/2 (**A**). In Caspase-3 testing, similar results were achieved, yet this time meeting statistical significance (**C**). One-way ANOVA with Tukey’s post hoc test was used for analysis of all experimental groups. SEM is presented alongside mean values. * *p* < 0.05; ** *p* < 0.01; *** *p* < 0.001.

At the signaling level, the combined pretreatment also downregulated all investigated proteins compared with the control cohort, albeit to different extents than tacrolimus monotherapy (Figure 7). ERK1/2 (−28.1%; *p* = 0.4287) and SAPK/JNK (−34.5%; *p* = 0.3017) showed non-significant but notable negative regulatory trends after IRI induction and combined pretreatment (Figure 7A,B). Caspase-3 and PARP were significantly reduced under this condition, with Caspase-3 decreasing by −59.2% (*p* < 0.001) and PARP by −80.1% (*p* = 0.0262) (Figure 7C,D). Compared with the tacrolimus group, these activity reductions were slightly more pronounced for ERK1/2 and PARP, yet not to a significant extent (ERK1/2: *p* = 0.9995; PARP: *p* = 0.8972; Figure 7A,D). For Caspase-3 and SAPK/JNK, tacrolimus monotherapy attenuated activity more strongly than the combined therapy, but again without statistical significance (Caspase-3: *p* = 0.9445; SAPK/JNK: *p* = 0.2350; Figure 7B,C).

Consistent with these redox and signaling findings, the combined therapy also led to a significant reduction in TUNEL-positive hepatic cell death of −62.3% compared with the control cohort (Figure 6B and Figure S2C; *p* < 0.001), without differing from tacrolimus monotherapy (*p* > 0.9999).

**Figure 8:**
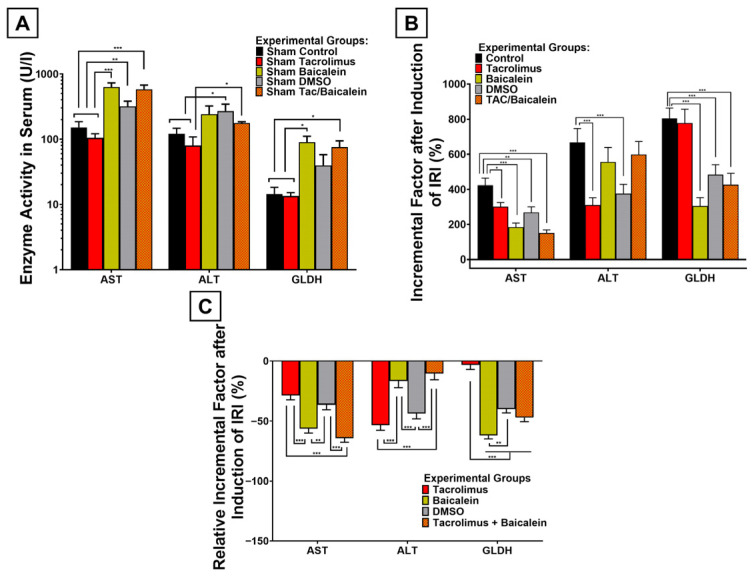
Comparative analyses between different pretreatments with regard to serum parameters displaying hepatic cell integrity, obtained by caval blood sampling. (**A**): Graphical representation of measured enzyme activity in serum after sham operation to demonstrate the sole effect of the respective treatment on hepatic cell integrity parameters compared to no treatment. Whereas tacrolimus treatment even led to a slight insignificant decrease in enzyme activity, the other three pretreatment cohorts (baicalein, DMSO, and TAC + baicalein) presented with mostly significant increases in liver enzymes, most notably in AST. (**B**): Parameter-specific incremental factors of the respective serum parameters after induction of IRI are shown as a quotient of enzymes measured after ischemia and reperfusion over the same enzyme measured after sham operation. Each pretreatment showed—mostly significant—measurable attenuation in liver enzyme increases in the serum after IRI as compared to the control cohort, most notably in AST. (**C**): These parameter-specific relative incremental factors after respective pretreatment and hepatic IRI of the respective serum parameters are shown as the relative difference in the respective incremental factors after the respective pretreatment compared to no pretreatment. Tacrolimus preconditioning was most effective in attenuating ALT increase, baicalein in GLDH and the combined therapy of both in attenuating AST increase. Two-way ANOVA with Šídák’s post hoc test was used for analysis of all experimental groups. SEM is presented alongside mean values. * *p* < 0.05; ** *p* < 0.01; *** *p* < 0.001.

When evaluating the potential toxicity of the combined therapy, this treatment led to a significant increase in enzyme release even in sham-operated mice, with AST increasing by +282.4% (*p* < 0.001) and GLDH by +417.2% (*p* = 0.02), whereas the +46.3% increase in ALT was not significant (*p* = 0.14) (Figure 8A). These increases were significantly higher than in the tacrolimus group, in which liver enzymes did not rise under sham conditions (AST: *p* < 0.001; ALT: *p* = 0.0373; GLDH: *p* = 0.0132) (Figure 8A). Nonetheless, the incremental factor by which IRI increased liver enzyme release could still be reduced by the combined treatment compared with the control cohort (Figure 8B). The AST increase was attenuated by −64.4% (*p* < 0.001), GLDH by −47.1% (*p* < 0.001), and ALT by only −10.4% (*p* = 0.5287). These relative incremental factors were significantly more negative than in the tacrolimus cohort for AST (2.23-fold) and GLDH (14.1-fold) (each *p* < 0.001) (Figure 8C). In contrast, tacrolimus outperformed the combined therapy for attenuation of ALT release after IRI induction by 5.11-fold (*p* < 0.001).

### 3.7. Comparative Analysis with Previous 12/15-LOX Inhibition Experiments

In a final step, we compared tacrolimus and the combined therapy of tacrolimus and baicalein with the previously published baicalein and carrier-solution (DMSO) cohorts from our working group [33]. At the upstream metabolic level, tacrolimus produced the numerically strongest increase in oxidative glutathione metabolism, although no significant differences were observed between tacrolimus, baicalein, and the combined treatment (tacrolimus vs. baicalein: *p* = 0.2732; baicalein vs. combined: *p* = 0.9307; tacrolimus vs. combined: *p* = 0.2431) (Figure 6B). At the signaling level, all principal treatment strategies shared the common pattern of reduced ERK1/2, SAPK/JNK, Caspase-3, and PARP activation (Figure 7). Although the magnitude of these effects differed slightly, tacrolimus and baicalein did not significantly differ from one another (ERK: *p* = 0.9923; SAPK/JNK: *p* > 0.999; Caspase-3: *p* = 0.2428; PARP: *p* = 0.9887), and the combined therapy showed the same directional pattern, albeit with less pronounced SAPK/JNK attenuation than baicalein (*p* = 0.2683) (Figure 7B). At the phenotypic level, TUNEL-positive hepatic cell death was comparably reduced by baicalein, tacrolimus, and the combined treatment (each *p* > 0.999 for between-treatment comparisons; each active treatment was significant vs. control: tacrolimus *p* = 0.0015, baicalein *p* < 0.001, and combined *p* < 0.001) (Figure 6A). A different pattern emerged for the serum injury markers (Figure 8): because the combined therapy shared the sham-related enzyme increases observed with baicalein (and DMSO), its incremental-factor reduction after IRI induction was more aligned with these cohorts than with the tacrolimus group (Figure 8B). Accordingly, tacrolimus attenuated the ALT increase more strongly than baicalein and the combined treatment (*p* < 0.001), whereas the latter two showed a significantly stronger reduction in AST and GLDH release after IRI than tacrolimus (each *p* < 0.001) (Figure 8C).

## 4. Discussion

Understanding and tackling hepatic IRI plays a pivotal role in both LTx and major liver resection [68,69,70]. The acute parenchymal damage induced by IRI at the onset of surgery can profoundly influence postoperative recovery and long-term outcomes [2,49,69,71]. In LTx, persistent organ shortage has led to the increasing use of marginal grafts, which are particularly susceptible to IRI stress and are associated with impaired graft and patient survival [72,73]. In hepatic surgery, the growing adoption of multimodal treatments and extensive resection strategies frequently necessitates temporary inflow occlusion, such as the Pringle maneuver, thereby predisposing the liver to ischemic insult and reperfusion-related injury [74,75]. Hence, gaining further insights into modifiable pathways that contribute to hepatic IRI is of critical importance, as these mechanisms represent potential therapeutic targets to mitigate perioperative liver injury.

With the present study, we demonstrated that tacrolimus preconditioning significantly reduces hepatocellular death in postischemic liver tissue. Importantly, our experimental design was intended to detect pathway-associated changes in vivo, not to establish a direct molecular interaction between tacrolimus and 12/15-LOX or GPx4. The current data therefore support a mechanistic association rather than direct target engagement: tacrolimus treatment coincided with changes in glutathione redox metabolism, attenuation of stress-responsive MAPK signaling, and reduced apoptotic execution, all of which involve pathways previously implicated in hepatic IRI and in our prior baicalein study [33]. Several non-mutually exclusive mechanisms may contribute to these cytoprotective effects. One relates to the well-established anti-inflammatory properties of tacrolimus: inflammation is a central component of hepatic IRI physiology [49]. Both Kupffer cells and CD4^+^ T-lymphocytes play key roles by releasing cytokines and thereby impairing microcirculation [76,77,78,79]. CD4^+^ T cells are also considered fundamental for the development of hepatic IRI, as they interact with Kupffer cells in addition to their independent effect, thereby increasing ROS formation [49,78,79]. As mentioned earlier, CD4^+^ T cells are effectively inhibited by tacrolimus [42,43,44,45,46,47,48]. These anti-inflammatory properties likely contribute to improved hepatic microcirculation via reduced endothelin-1 release, less ROS generation by suppression of Kupffer cell activation, and attenuation of oxidative damage by specifically blocking mitochondrial membrane transition [15,80,81]. To date, most investigations into the hepatoprotective potential of tacrolimus have emphasized these general anti-inflammatory and anti-oxidative effects.

In the present study, tacrolimus was associated with a significant increase in the GSSG/GSH quotient, which we interpret as an indirect sign of altered oxidative glutathione turnover. This observation aligns with our previous results identifying glutathione homeostasis as a key determinant in hepatic IRI [33]. However, because neither GPx4 protein abundance/activity nor 12/15-LOX activity was measured directly, the present data do not prove that tacrolimus directly targets either protein. Rather, they are compatible with an upstream effect on the glutathione-dependent redox environment in which GPx4 operates, as existing evidence strongly suggests that its activity represents a principal contributor to the observed upregulation of glutathione-dependent oxidation. This interpretation is biologically plausible as GPx4 plays a pivotal role in hepatic IRI as an enzymatic antagonist of 12/15-LOX, which catalyzes the peroxidation of membrane-bound fatty acids and thereby compromises cellular integrity [17,20]. The 12/15-LOX product 12S/15S-HpETE has been identified as an early lipid peroxidate that initiates cell death and serves as a central source of oxidative stress after IRI in vitro, which is presumably due to the mitochondrial location of the metabolite [17,19,20]. The location in the mitochondrial membrane of these metabolites therefore suggests that the protective effect of glutathione oxidation is primarily caused by GPx4, as this is the only GPx that can metabolize membrane-bound lipid hydroperoxides [17,19,20]. When investigating hepatic IRI, it is imperative to acknowledge GPx4 as a pivotal factor in the survival of hepatocytes in both in vitro and in vivo experiments [27]. A decrease in mitochondrial-induced apoptosis has therefore already been attributed to Gpx4 [17]. In this context, it is most likely assumed that the reduced occurrence of lipid hydroperoxides mediated by GPx4 leads to reduced cytochrome C release [26,28]. Mitochondrial GSH (reduced glutathione), which accounts for approximately 15% of the total GSH reservoir in the human organism, has already been identified as a relevant antioxidant for cell survival [29]. In this context, our previous baicalein study is instructive: pharmacological 12/15-LOX inhibition enhanced GPx-related glutathione metabolism and modulated the same downstream mediators investigated here [33]. Taken together, these findings suggest convergence on a common redox-sensitive injury axis, while stopping short of establishing direct molecular regulation by tacrolimus.

Based on prior studies investigating the interplay of 12/15-LOX and GPx4, mitochondrial integrity appears to represent a central determinant of hepatic IRI at the molecular level [16,17,18,19,20]. The present findings suggest that tacrolimus preconditioning might preserve this integrity, thereby mitigating subsequent cellular injury [16,17,18,19,20]. While hepatic IRI was long considered to be dominated by regulated, cascade-dependent apoptosis, more recent evidence indicates that necrosis and intermediate forms of cell death substantially contribute to tissue damage as well [15,82]. Again, the mitochondrion might play a crucial role in shifting cell death response from one form of cell death to another by effecting mechanisms such as mitochondrial membrane transition [15,82]. The proportion of necrosis in hepatic IRI also explains the inflammatory response that apoptosis does not exhibit to the same extent: rupture of the plasma membrane releases intracellular components such as alarmins and so-called DAMPs (damage-associated patterns), which in turn activate a systemic inflammatory response [83]. This also elucidates the positive effects of T-cell-associated tacrolimus therapy. Furthermore, ferroptosis has emerged as a relevant mode of cell death in hepatic IRI. In particular, the role of Gpx4 in ferroptotic cell death has been the subject of scientific scrutiny on multiple occasions in recent years [84,85,86]. Baicalein, which was used to inhibit 12/15-LOX activity in previous experiments, is known to specifically inhibit ferroptosis in experimental settings [33,87]. The overlap between the current tacrolimus phenotype and our prior baicalein phenotype is therefore consistent with the possibility that tacrolimus dampens lipid peroxidation-driven injury signaling and that consequently, ferroptosis might be assumed to play a major role after tacrolimus preconditioning as well. Yet this remains an inference. In addition, research at the hepatocellular level has demonstrated that Gpx4 can slow down and partly impede degenerative processes [27]. Consequently, the present study hypothesizes that both necrotic and ferroptotic cell death might be attenuated by successful intervention in the GPx4-associated injury network, alongside apoptosis.

Experimental liver transplant studies in rats, in which donor grafts were flushed ex vivo with tacrolimus, have previously suggested a link between tacrolimus and glutathione metabolism in postischemic livers, although the underlying mechanisms remained undefined [59]. Building upon these findings, subsequent studies—including a human trial conducted in our department—evaluated the effect of extracorporeal tacrolimus flushing of donor livers immediately prior to transplantation [43]. However, this trial was unable to demonstrate significant beneficial effects, presumably due to its study design involving marginal organs only and the lack of genuine donor preconditioning [88]. In light of the present results, we hypothesize that longer-term tacrolimus exposure under (pseudo-)physiological conditions could provide a more effective form of graft preconditioning, potentially translating the pathway-associated protective signals observed in this study into clinical practice. Meanwhile, normothermic machine perfusion, which mimics those physiological settings, is well established in many transplant centers and provides a valuable platform for therapeutic trials in grafts [89]. Respective trial protocols are currently being planned.

Our study demonstrated a significant reduction in several key proteins associated with stress signaling and apoptotic execution in hepatic IRI and in our previous 12/15-LOX-associated experiments [33]. Most notably, the activities of SAPK/JNK, Caspase-3 and PARP were markedly decreased following tacrolimus preconditioning. ERK1/2 was also downregulated, albeit to a lesser and statistically non-significant extent, and may therefore have contributed to the attenuated cell death response. ERK1/2 phosphorylation is known to occur rapidly, i.e., approx. 20 min after a stress trigger, and to decline thereafter [34]. Consequently, the study protocol, which utilized tissue obtained 90 min following the presumed trigger for oxidative stress, namely reperfusion, might potentially encompass a duration that is overly protracted to capture a stronger ERK1/2 signal. The fact that attenuation was still measurable in this confounder-prone in vivo setting nonetheless suggests a certain degree of biological relevance. Also, as ERK1/2 is known to be affected by multiple external stressors and is an effector protein that is situated rather upstream in a stress-response scenario, the general anti-oxidative properties of tacrolimus might be influential as well [35]. At the same time, these mediator changes should be interpreted as downstream correlates of the injury response rather than proof of a direct linear chain initiated by tacrolimus. SAPK/JNK is a recognized indicator of oxidative cell stress [36], and Caspase-3 is known as a key effector mediating cell death in hepatic IRI and was shown to be of relevance in other experiments observing tacrolimus-associated cell death attenuation as well [37,38]. PARP is directly mediated by Caspase-3; thus, its cleavage reflects downstream execution signaling [39]. Their ordered modulation together with the glutathione readout therefore supports a coherent pathway model in which upstream redox changes are accompanied by attenuation of MAPK signaling and subsequent apoptotic execution. This fosters the impression of relevance for this injury network cascade in hepatic IRI, as described in previous studies [33], as well as provides insights into potential beneficial modes of action of tacrolimus.

This cautious interpretation is in line with the current literature. Tacrolimus is primarily understood to exert its canonical biological effects through binding to FKBP12 and formation of an inhibitory complex with calcineurin [90,91]. At the same time, recent studies in hepatic ischemia–reperfusion injury indicate that GPX4-associated ferroptotic susceptibility can be regulated at multiple upstream levels, including GPX4 promoter methylation [92], ACSL4-dependent lipid remodeling [93], and insulin-induced gene 2-dependent control of GPX4 expression [94]. Against this background, the present findings are best interpreted as indirect evidence that tacrolimus modulates a 12/15-LOX/GPx4-associated injury network at the pathway level, rather than as proof of direct molecular interaction with 12/15-LOX or GPX4. The coordinated preservation of glutathione-related redox homeostasis together with attenuation of SAPK/JNK-, ERK1/2-, Caspase-3-, and PARP-associated injury signaling is consistent with such a network-based interpretation.

When compared to our previous findings with the sole use of baicalein and DMSO, tacrolimus possesses a major advantage which is its lack of hepatotoxic properties [33]. The dosage of tacrolimus used in this experiment (300 µg/kg body weight) equals a dosage that would be applicable in human settings, but with an elevated risk for, e.g., nephrotoxic or neurotoxic side effects, as the average dose in human settings is considerably lower (50 µg/kg body weight). However, the dosage was chosen deliberately to be rather high in order to visualize a drug effect in this proof-of-principle setting. Despite the applied dose, there was no evident hepatotoxic effect as no elevated serum liver enzymes were detected as compared to the control cohort, in both sham and intervention groups. After induction of IRI, tacrolimus preconditioning led to a significant reduction in the expected rise in liver enzymes, representing the IRI-attenuating features of tacrolimus. The highest impact was proven for ALT which is the most liver-specific of the aforementioned enzymes [95]. Baicalein, dissolved in DMSO, which was also dosed in a highly elevated fashion, showed comparable results, although partially less pronounced than with tacrolimus [33]. However, there was a marked increase in liver enzymes during the sham operation, representing hepatotoxicity of the drugs administered [33]. This was yet again attenuated after IRI induction due to its protective effect in postischemic settings; also, this attenuation was mostly expressed within the less liver-specific enzymes AST and GLDH [95]. Hence, one could suggest that tacrolimus is a lot less toxic to the liver and shows more liver-specific effects. Another major advantage of tacrolimus is that it is the main and primary immunosuppressant drug already used for liver transplantation worldwide [96]. There is vast experience with its application, the different modes of dosing it, and how it is metabolized in the complex hemostasis of an LTx recipient [96]. Therefore, the translational aspect of applying insights gained from this study is a major step closer to real-life settings than with other drugs, i.e., baicalein.

Furthermore, combined baicalein and tacrolimus pretreatment did not amplify any analyzed parameter relative to either monotherapy. In contrast, the toxic effects specific to the baicalein/DMSO groups were again observed, thereby attenuating the otherwise beneficial results of the tacrolimus mono cohort. The lack of additivity may indicate partial pathway convergence or a ceiling effect within the measured readouts. This would explain the lack of amplification within the observed results. However, our design does not allow us to discriminate between these possibilities, and it should therefore not be taken as proof that tacrolimus and baicalein act through identical mechanisms.

It needs to be pointed out that this study features various limitations. Most importantly, it was designed as an in vivo proof-of-principle experiment and not as a target-engagement study. We did not assess direct binding of tacrolimus to 12/15-LOX or GPx4, nor did we quantify 12/15-LOX activity, GPx4 protein abundance or enzymatic activity, lipid peroxidation intermediates, mitochondrial readouts, or genetic/pharmacological rescue approaches. Therefore, causality within the proposed redox–signaling–cell death axis cannot be definitively assigned, and our mechanistic interpretation remains inferential. Also, the study does not prove that tacrolimus preserves mitochondrial integrity or prevents ferroptosis per se. Rather than proving direct molecular regulation, our data identify a coherent pattern of upstream metabolic and downstream signaling changes that is consistent with modulation of a 12/15-LOX/GPx4-associated injury pathway and compatible with reduced oxidative stress-mediated cell death propagation. As this was designed as a proof-of-principle experiment, the dose for preconditioning was deliberately chosen to be high in order to visualize a drug effect in the liver. It remains uncertain whether doses optimized for systemic safety, particularly regarding kidneys and nerves, would lead to the same effect in a single-dose experiment. On the other hand, one could argue that preconditioning of a liver graft could now be performed via normothermic machine perfusion which would allow for higher local dosages without simultaneous exposure of other organ systems. Furthermore, although most of our findings reach statistical significance, the total number of involved experimental animals was limited to *n* = 10 per group in order to comply with current guidelines for laboratory animal protection and avoid unnecessary use of live animals. Due to the significant results achieved and clear-cut conclusions drawn already at this cohort size, we are confident that the number of animals used was justified. Another limitation aspect relates to the experimental time course. We deliberately chose a rather short setting of 60 min of ischemia and 90 min of reperfusion to visualize early pathway changes as a proof of principle. Longer periods might have produced more pronounced injury patterns but would also have increased the risk of masking short-term signaling events such as ERK1/2 or introducing additional systemic confounders. To scrutinize these assumptions, further mechanistic and time-course studies would be required.

## 5. Conclusions

In conclusion, tacrolimus preconditioning significantly attenuates hepatocellular death following hepatic ischemia and reperfusion in this model. Tacrolimus treatment was associated with altered oxidative glutathione metabolism, attenuation of stress-responsive ERK1/2 and SAPK/JNK signaling, and reduced activation of apoptotic mediators, including PARP and Caspase-3. These findings are consistent with modulation of a 12/15-LOX/GPx4-associated redox–signaling axis, yet this inference bears certain caveats as no direct molecular regulation by tacrolimus was proven in this study. Tacrolimus administration did not induce evident hepatotoxic effects and was associated with a markedly attenuated rise in serum liver enzymes after IRI induction. Given that tacrolimus is already the immunosuppressant of choice in liver transplantation, these observations support further mechanistic and translational studies and may hold translational potential through evaluation in graft-preconditioning settings such as normothermic machine perfusion. If comparable results are observed in these settings, this could represent a feasible strategy to further reduce ischemia–reperfusion injury and improve long-term transplant outcomes.

## Data Availability

The original contributions presented in this study are included in the article/Supplementary Materials. Further inquiries can be directed to the corresponding author.

## Supplementary Materials

The following supporting information can be downloaded at: https://www.mdpi.com/article/10.3390/antiox15050557/s1. Figure S1. Uncropped images of Western Blot assays of proapoptotic mediator proteins ERK 1/2 (Mitogen-activated protein kinase p44/42, 42/44 kDa, (A), SAPK/JNK (stress-activated protein kinase/Jun-amino-terminal kinase, 46/54 kDa, (B), Caspase-3 (17/19 (cleaved)/35 (total) kDa, (C) and PARP (poly-ADP-ribose-polymerase, 89 (cleaved)/116 (total) kDa, (D), obtained and subsequently analyzed from liver tissue (right anterior segment) after inducing ischemia and reperfusion, without pretreatment (I), with Tacrolimus preconditioning (II) and combined preconditioning with Baicalein and Tacrolimus (III). For each protein, seven lanes were incubated and analyzed per membrane. The first lane comes corresponds to a molecular weight standard, lanes that do not correspond to experimental group blots (i.e., negative & positive controls) are marked with an X. Both the activated (1) subvariant and the total variant (2) of each protein were assessed on the same membranes by sequential probing with stripping between incubations. Activated subvariants are defined as phosphorylated forms for ERK1/2 (A1) and SAPK/JNK (B1), and cleaved forms for Caspase-3 (C1) and PARP (D). For PARP, both variants are blotted on one membrane: The upper variant at 116 kDa represents the total form, the lower variant at 89 kDa represents the cleaved form. Figure S2. Evaluation of cell death proportion within liver tissue after induction of hepatic ischemia-reperfusion-injury. Representative images of fluorescence microscopic detection of decayed cells of the investigated liver sample (left posterior segment) using TUNEL assays, without pretreatment (A), with Tacrolimus preconditioning (B) and combined preconditioning with Baicalein and Tacrolimus (C). The following parameters were utilized: 10x enlargement and an emission wavelength of 450–500 nm and a fluorescence detection range of 515–565 nm (green light). TUNEL-positive cell nuclei are marked as fluorescent green.

## Abbreviations

The following abbreviations are used in this manuscript:

°C	Degrees centigrade
µg/kg	Micrograms/kilogram
12/15-LOX	12/15-lipoxygenase
12S/15S-HpETE	12S/15S-hydroperoxyeicosatetraenoic acid
ALT	Alanine aminotransferase
ANOVA	Analysis of variance
AIF	Apoptosis-inducing factor
AST	Aspartate aminotransferase
ATP	Adenosine triphosphate
CD	Cluster of differentiation
DAMP	Damage-associated pattern
DAPI	4′,6-Diamidino-2-phenylindole
DTT	Dithiothreitol
DMSO	Dimethyl sulfoxide
DTNB	5,5-Dithiobisnitrobenzoeic acid
ERK1/2	Mitogen-activated protein kinase p44/42
FK506/TAC	Tacrolimus
GLDH	Glyceraldehyde-3-phosphate dehydrogenase
GPx4	Glutathione peroxidase 4
GAPDH	Glyceraldehyde-3-phosphate dehydrogenase
GSH	(Reduced) glutathione
GSSG	(Oxidized) glutathione disulfide
h	Hours
HRP	Horseradish peroxide
IgG	Immunoglobulin G
IRI	Ischemia–reperfusion injury
kDa	Kilodalton
LOCI	Laboratory for Optical and Computational Instrumentation
LTx	Liver transplantation
MAP	Mean arterial blood pressure
min	Minutes
mmHg	Millimeters mercury
n	Number
NEM	N-ethylmaleimide
PARP	Poly-ADP-ribose-polymerase
ROI	Region of interest
ROS	Reactive oxygen species
SAPK/JNK	Stress-activated protein kinase/Jun-amino-terminal kinase
SEM	Standard error of mean
TNB	Thionitrobenzoeic acid
TUNEL	Terminal deoxynucleotidyl transferase dUTP nick end labeling
USA	United States of America
xg	Times gravity
