# Supplementary material for: Tacrolimus (FK506) Attenuates Hepatic Ischemia–Reperfusion Injury via Oxidative Glutathione Metabolism and Suppression of Lipoxygenase-Mediated Cell Death
Source: Antioxidants (Basel). 2026 Apr 28;15(5):557. doi: 10.3390/antiox15050557 (PMC13203124; doi:10.3390/antiox15050557)
Supplement: Supplementary file 1 [file antioxidants-15-00557-s001.zip › antioxidants-4224716-supplementary.pdf]

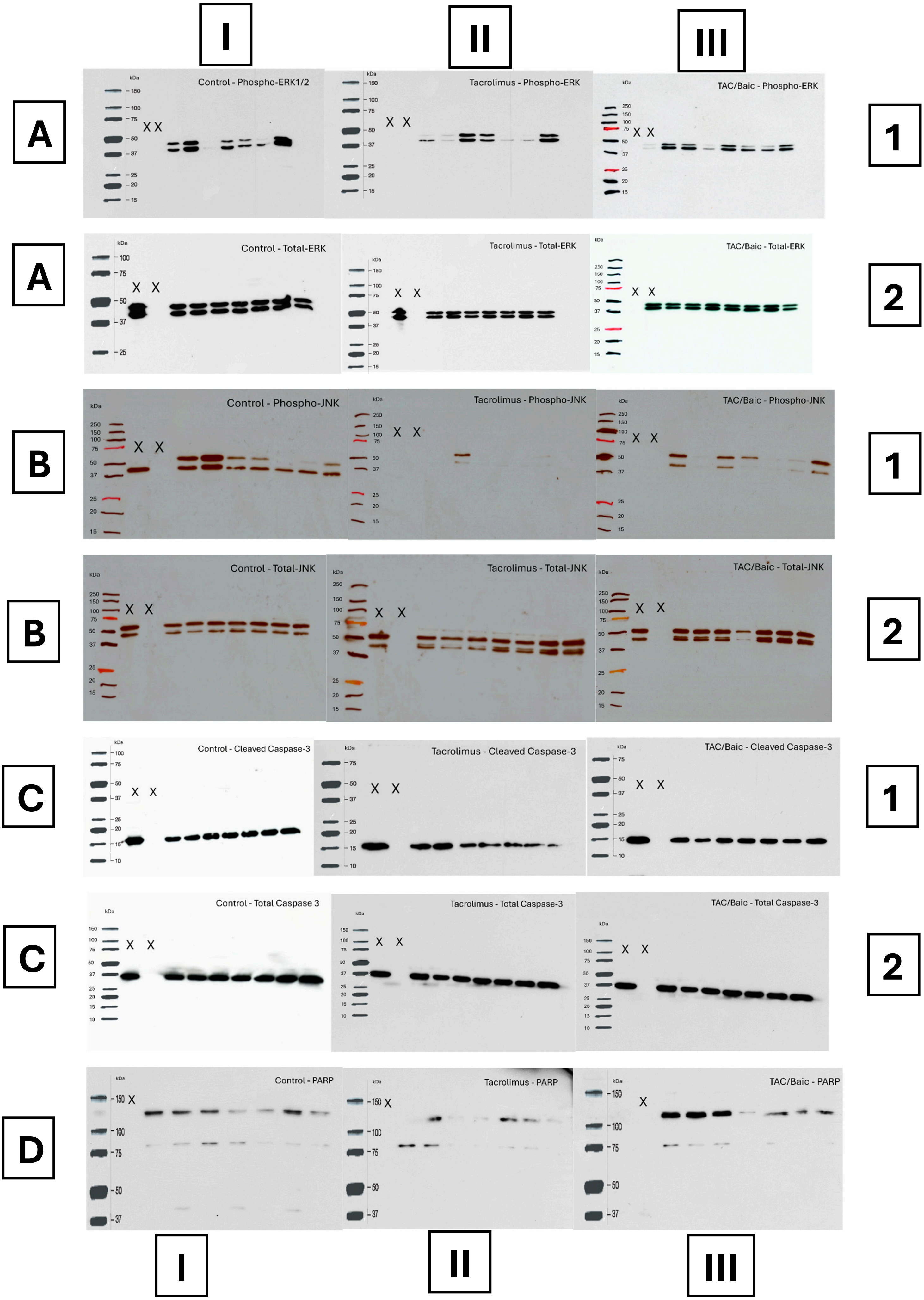

I
II
III
A
Control - Phospho-ERK1/2
Tacrolimus - Phospho-ERK
TAC/Baic - Phospho-ERK
1
A
Control - Total-ERK
Tacrolimus - Total-ERK
TAC/Baic - Total-ERK
2
B
Control - Phospho-JNK
Tacrolimus - Phospho-JNK
TAC/Baic - Phospho-JNK
1
B
Control - Total-JNK
Tacrolimus - Total-JNK
TAC/Baic - Total-JNK
2
C
Control - Cleaved Caspase-3
Tacrolimus - Cleaved Caspase-3
TAC/Baic - Cleaved Caspase-3
1
C
Control - Total Caspase 3
Tacrolimus - Total Caspase-3
TAC/Baic - Total Caspase-3
2
D
Control - PARP
Tacrolimus - PARP
TAC/Baic - PARP
I
II
III

Figure S1. Uncropped images of Western Blot assays of proapoptotic mediator proteins ERK 1/2 (Mitogen-activated protein kinase p44/42, 42/44 kDa, A), SAPK/JNK (stress-activated protein kinase/Jun-amino-terminal kinase, 46/54 kDa, B), Caspase-3 (17/19 (cleaved) / 35 (total) kDa, C) and PARP (poly-ADP-ribose-polymerase, 89 (cleaved) /116 (total) kDa, D), obtained and subsequently analyzed from liver tissue (right anterior segment) after inducing ischemia and reperfusion, without pretreatment (I), with Tacrolimus preconditioning (II) and combined preconditioning with Baicalein and Tacrolimus (III). For each protein, seven lanes were incubated and analyzed per membrane. The first lane comes corresponds to a molecular weight standard, lanes that do not correspond to experimental group blots (i.e. negative & positive controls) are marked with an X. Both the activated (1) subvariant and the total variant (2) of each protein were assessed on the same membranes by sequential probing with stripping between incubations. Activated subvariants are defined as phosphorylated forms for ERK1/2 (A1) and SAPK/JNK (B1), and cleaved forms for Caspase-3 (C1) and PARP (D). For PARP, both variants are blotted on one membrane: The upper variant at 116 kDa represents the total form, the lower variant at 89 kDa represents the cleaved form.

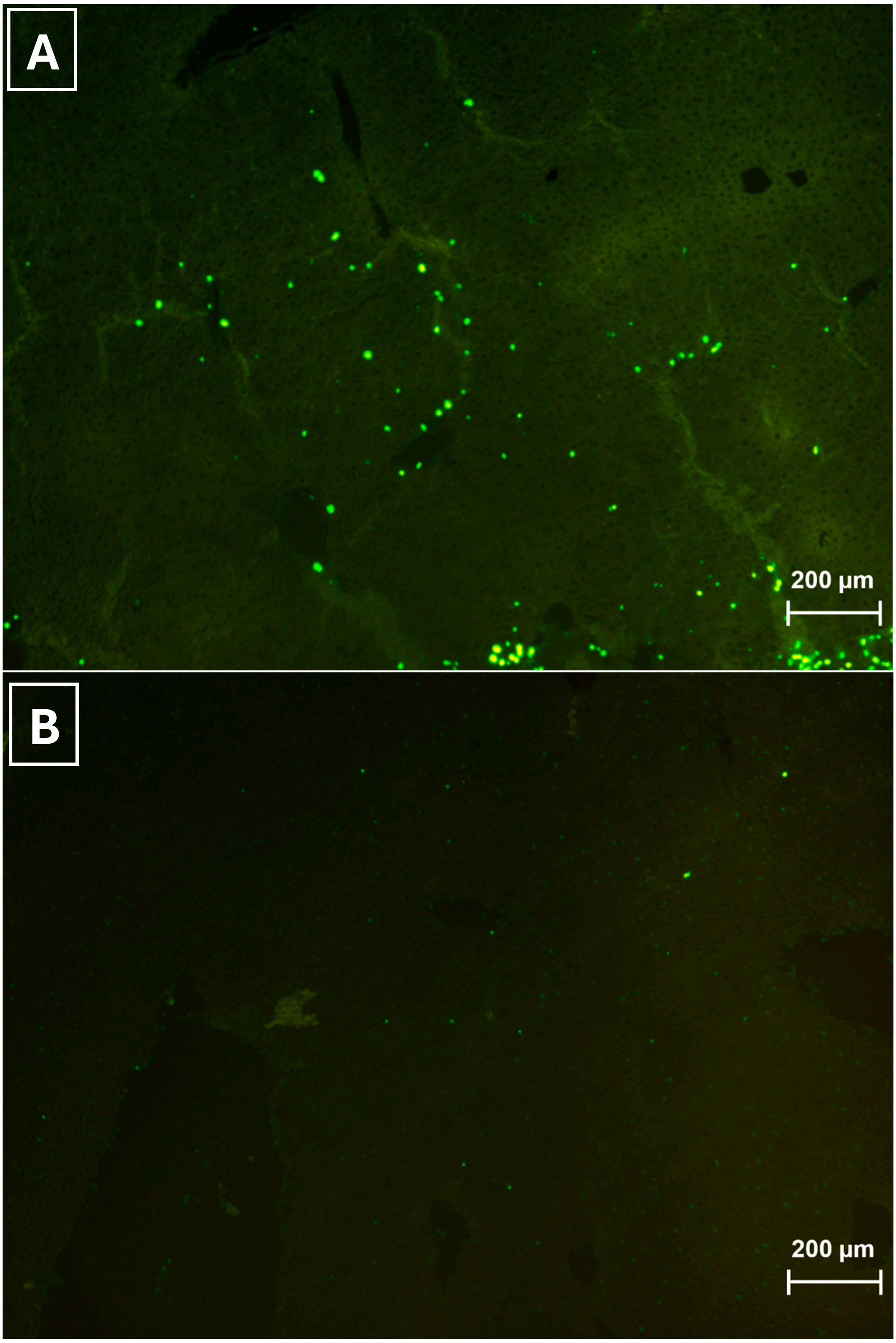
A
200 µm
B
200 µm

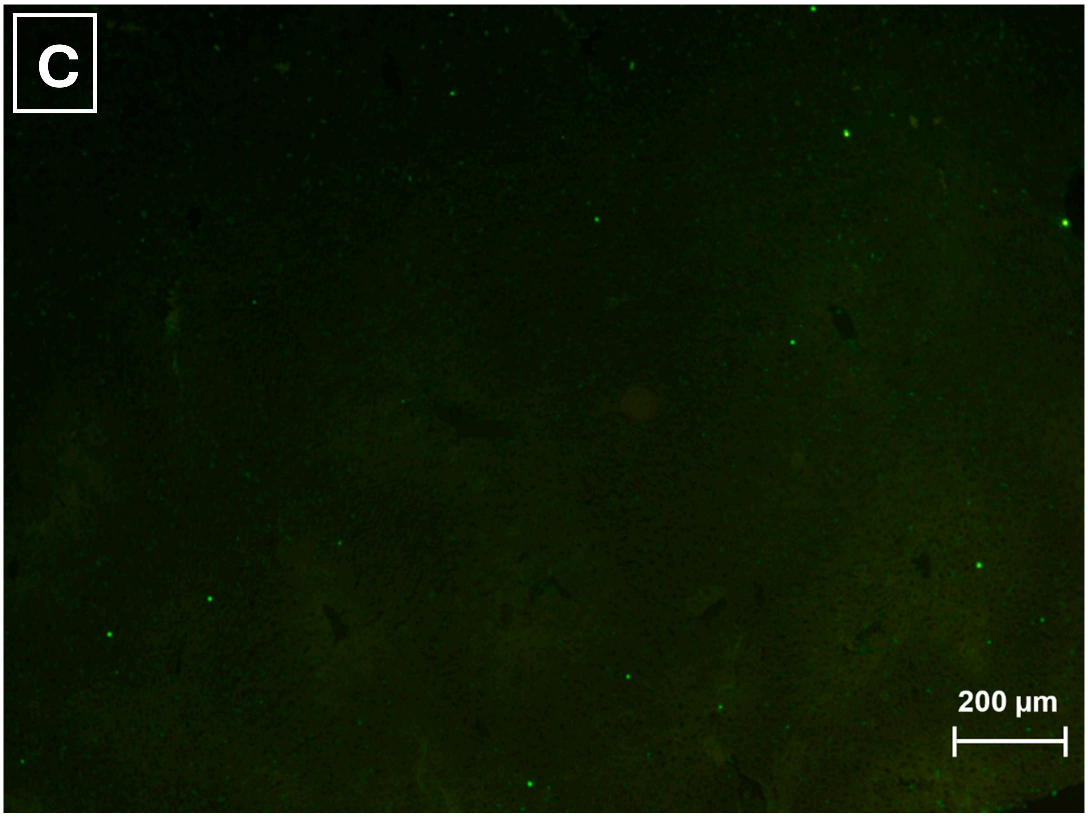

Figure S2. Evaluation of cell death proportion within liver tissue after induction of hepatic ischemia-reperfusion-injury. Representative images of fluorescence microscopic detection of decayed cells of the investigated liver sample (left posterior segment) using TUNEL assays, without pretreatment (A), with Tacrolimus preconditioning (B) and combined preconditioning with Baicalein and Tacrolimus (C). The following parameters were utilized: 10x enlargement and an emission wavelength of 450–500 nm and a fluorescence detection range of 515–565 nm (green light). TUNEL-positive cell nuclei are marked as fluorescent green.
